# Analysis of the 2017-2018 Rift valley fever outbreak in Yirol East County, South Sudan: a one health perspective

**DOI:** 10.11604/pamj.supp.2022.42.1.33769

**Published:** 2022-06-09

**Authors:** Otim Patrick Cossy Ramadan, Kibebu Kinfu Berta, Joseph Francis Wamala, Sylvester Maleghemi, John Rumunu, Caroline Ryan, Alice Igale Ladu, Julu Louis Kenyi Joseph, Abraham Aduet Abenego, Fabian Ndenzako, Olushayo Oluseun Olu

**Affiliations:** 1 World Health Organization, East and South Africa, Sub Regional Office, Nairobi, Kenya,; 2 World Health Organization (WHO), WHO Country Office, Juba, South Sudan,; 3 Ministry of Health, Juba, Republic of South Sudan

**Keywords:** Rift Valley fever (RVF), zoonotic disease, surveillance, one health, South Sudan

## Abstract

**Introduction:**

the emergence and re-emergence of zoonotic diseases have threatened both human and animal health globally since their identification in the 20th century. Rift Valley fever (RVF) virus is a recurrent zoonotic disease in South Sudan, with the earliest RVF cases confirmed in 2007 in Kapoeta North County, Eastern Equatoria state.

**Methods:**

we analyzed national RVF outbreak data to describe the epidemiological pattern of the RVF outbreak in Yirol East county in Lakes State. The line list of cases (confirmed, probable, suspected, and non-cases) was used to describe the pattern and risk factors associated with the outbreak. The animal and human blood samples were tested using Enzyme-Linked Immunosorbent Assay (ELISA) (Immunoglobulin IgG and IgM) and Reverse Transcriptase-Polymerase Chain Reaction (RT-PCR). Qualitative data were collected from weekly RVF situation reports, and national guidelines and policies.

**Results:**

between December 2017 and December 2018, 58 suspected human RVF cases were reported. The cases were reclassified based on laboratory and investigations results, such that as of 16th December 2018, there were a total of six (10.3%) laboratory-confirmed, three (5.2%) probable, one (1.7%) suspected, and 48 (82.8%) non-cases were reported. A total of four deaths were reported during the outbreak (case fatality rate (CFR) 6.8% (4/58). A total of 28 samples were collected from animals; of these, six tested positives for RVF (positivity rate of 32.1% (9/28). The outbreak was announced in March 2018, after four months of the first reported suspected RVF case. Several factors were attributed to the delayed notification and outbreak announcement such as lack of multi-sectorial coordination at the state and county level, multi-sectoral coordination at national level mostly attended by public health experts from human health, inadequate animal health surveillance, poor coordination between livestock disease surveillance and public health surveillance, limited in-country laboratory diagnostic capacity, the laboratory results for the animal health took longer than expected, and lack of a national One Health approach strategy.

**Conclusion:**

the outbreak demonstrated gaps to investigate and respond to zoonotic disease outbreaks in South Sudan.

## Introduction

There has been an increased global interest in emerging zoonotic diseases [1]. Zoonotic diseases are: “any diseases or infections that are naturally transmissible from vertebrate animals to humans” [1]. There are over 200 known types of zoonoses; they comprise a large proportion of existing and new diseases in humans [1]. Rift Valley fever (RVF) is one such zoonotic disease that is caused by the Rift valley fever virus (RVFV) [2]. Rift valley fever virus is in the Phlebovirus genus, order of Bunyavirales, family Phenuiviridae first identified in 1931 [3]. Rift valley fever virus is an acute arthropod-borne viral disease that primarily affects wild and domestic ruminants including cattle, goats, sheep, buffalos, and camels and humans. Infection can cause severe diseases in both animals and humans and because of long inter epi-zoonotic intervals is referred to as a re-emerging disease. Most human infections result from direct or indirect contact with the organs or blood of infected animals [4]. The virus can be transmitted to humans by handling animal tissue during slaughtering or butchering, assisting with animal births, conducting veterinary procedures, or from the disposal of carcasses or fetuses [5]. Human infection also occurs from the bites of infected mosquitos, most commonly Aedes, Anopheles and Culex species, and transmission through blood-feeding flies; however, there are no reported cases of human-to-human transmission to date [5]. Rift valley fever virus causes a storm of abortions in domestic and wild pregnant animals and excess mortality among young animals [6-8]. The disease in humans causes severe forms of “influenza-like” infection characterized by headache, fever, vomiting, weight loss, and muscle pain with a mortality rate of <2% [9,10].

Since the first outbreak was detected among sheep in a farm in the Rift valley farm of Kenya in 1931, several outbreaks have been reported in sub-Saharan Africa, including Sudan [5]. In 2000, the virus spread outside of Africa, with the first case of RVF reported in Yemen and Saudi Arabia following infected livestock trade from Africa. This had raised concerns that the disease could extend to Europe and other parts of Asia [11]. As a result, there was a disruption of livestock trade between countries. Between 2006 and 2007, there was a 60 million US dollar economic loss experienced by East Africa countries due to the disruption of livestock trading [12]. Rift valley fever outbreak is a recurrent zoonotic disease in South Sudan. The outbreaks of RVF occurred in White Nile State (in Sudan) in 2007. This area borders the Upper Nile of South Sudan. A total of 747 confirmed human cases including 230 deaths (case fatality 30.8%) were reported, although it has been estimated 75,000 were infected [13,14]. During the 2007 RVF outbreak in Sudan, two suspect RVF deaths were reported from Renk and Jouda in South Sudan with heavy flooding in the whole of Upper Nile [13,15]. During the same year (2007) a suspect RVF human case was reported from Kapoeta North County and a follow-up animal serosurvey showed a prevalence of 0.9% for RVF IgM [16,17]. This study describes the epidemiology of the RVF outbreak in Yirol East County, South Sudan and presents the information generated during this outbreak. The study objectives are fourfold: first to understand the epidemiology of RVF outbreak that occurred between 2017-2018; second to examine the country´s laboratory capacity to test RVF; third to identify barriers to application of the One Health approach in investigating zoonosis; and fourth to develop recommendations to strengthen One Health approach n South Sudan.

## Methods

**Study setting:** the Republic of South Sudan is the youngest country in Africa, with a landmass of 619,745 km^2^ with an estimated population of 11.4 million [18]. The country is bordered by the Democratic Republic of Congo (DRC) and Uganda in the South, the Central Africa Republic in the West, Sudan in the North, Ethiopia in the East, and Kenya in the Southeast. It is subdivided into 10 States, three special administrative areas, and 80 administrative Counties [19]. Yirol East and livelihoods. The three main economic activities in Yirol East County are cattle keeping, the county is located 418 kilometers northwest of the capital Juba, in Lakes state in central South Sudan. The population estimate of the county was over 100, 000 based on a 2008 population census. The county is surrounded by lakes and rivers, which supply water for humans, livestock, and wildlife. Fish from these water bodies contribute a large part to the communities´ diet in fishing, and cultivation of crops. The outbreak primarily affected the Payams of Yali (particularly Wunthou Boma (also called Thonabutkok Village) and Pagarau. In the county, rainfall and flooding before the outbreak created pools of stagnant water, potentially enabling transovarially infected mosquito eggs (known to survive long periods on the soil surface) to hatch and amplify [20]. The surface water provided a breeding ground for the amplification of the arthropod vectors in an area hosting a large number of cattle communities. These Payams are within proximity to larger water bodies, including the White Nile, Wunthou River and Lake Yirol that serve as common water sources for humans, wild and domesticated livestock.

**The surveillance system for RVF in South Sudan:** the South Sudan disease surveillance uses the Integrated Disease Surveillance and Response (IDSR) and Early Warning and Response Network (EWARN). The IDSR strategy is designed to capture various diseases, including diseases of outbreak potential. RVF is one of the epidemic-prone diseases stated under the national IDSR Technical Guideline (2013). Accordingly, during the 2017/2018 RVF outbreak, the case definition in the National IDSR Technical guidelines was used for the field investigation. The guidelines define a suscpected RVF case depending on whether it is early or late presentation. The criteria include: *early disease:* acute febrile illness (axillary temperature >37.5°C or oral temperature of >38.0°C) of more than 48 hours duration that does not respond to antibiotics or antimalarial therapy, and is associated with: direct contact with sick or dead animal or its products AND/OR; recent travel (during last week) to, or living in an area where, after heavy rains, livestock die or abort, and where RVF virus activity is suspected/confirmed AND/OR; abrupt onset of any 1 or more of the following: exhaustion, backache, muscle pains, headache (often severe), discomfort when exposed to light, and nausea/vomiting AND/OR; nausea/vomiting, diarrhoea, or abdominal pain with 1 or more of the following: severe pallor: low platelets (thrombocytopenia) as evidence by the presence of small skin and mucous membrane hemorrhages. AND/OR: evidence of bleeding into the skin, bleeding from puncture wounds, from mucous membranes or nose, from the gastrointestinal tract and unnatural bleeding from the vagina AND/OR: clinical jaundice. *Late stages of diseases or complications (2-3 weeks after onset):* Patient who have experienced, in the preceding months a flu-like illness, with clinical criteria, who additionally develop the following: CNS manifestations which resemble meningoencephalitis AND/OR; unexplained visual loss OR; unexplained death following the sudden onset of acute flu-like illness with hemorrhage, meningoencephalitis, or visual loss during the preceding month. The guidelines incorporated several RVF surveillance tools for use in the health facility and community. The tools are case definition, alert identification, and blood sample collection, storage, and transportation procedures. Further, the country surveillance system incorporated an Early Warning Alert and Response System (EWARS); the system is designed to automatically flag alerts daily or weekly, inform surveillance officers and relevant authorities to verify and investigate the alert without delay.

**Outbreak detection and investigation:** in the epidemiological week 52 of 2017, the State Ministry of Health was notified of a cluster of deaths in the Thonabutkok village of Yali Payam. Three deceased persons were reported to have presented with hemorrhagic symptoms. A multidisciplinary national Rapid Response Team (RRT) was dispatched to support the state in conducting investigations. The team conducted interviews with the affected families, took verbal autopsies using the WHO standard tool and collected both human and animal blood samples [21]. The team witnessed zoonotic events such as abortion among sheep and goats, as well as carcasses of domestic animals that had recently died. Besides, the finding from the field investigation revealed that abortions in small animals (goats and sheep) occurred two months before human cases [22].

**Sample collection and testing procedure:** five milliliters of blood were collected from all patients with symptoms compatible with the suspect case definition. These samples were transported by road to Rumbek town, flown to Juba, and then to the Uganda Virus Research Institute (UVRI). Samples were tested for the common viral hemorrhagic fevers (i.e. Ebola, Marburg, Crimean Congo Hemorrhagic Fever (CCHF), yellow fever, and Sosuga virus) using reverse transcriptase-polymerase chain reaction (RT-PCR). Additionally, antibody tests using enzyme-linked immunosorbent assay (ELISA) were conducted. The ELISA tests confirm infection with the RVFV by showing the presence of IgM antibodies, which appear briefly to show an acute infection, and or IgG antibodies, which appear for an extended period. Both IgM and IgG antibodies are specific to the RVFV infection [23]. A confirmed case was defined as detecting Ribonucleic Acid (RNA) amplification, or IgM and IgG against RVF in both animal and human blood samples [23]. Twenty-eight livestock samples were also collected and transferred to Juba. The samples were shipped to the United Nations Food and Agricultural Organization (FAO) collaborating laboratory centre in South Africa and Uganda Viral Research Institute (UVRI) in Uganda.

**Data collection and analyses:** the quantitative data were collected from an Excel based RVF line list. All the 58 RVF suspected cases listed in the line list were included for the quantitative study. For qualitative data we have used weekly RVF situational report, weekly Integrated Disease Surveillance and Response (IDSR) report, South Sudan Joint External Evaluation (JEE) of core capacity for International Health Regulation (IHR 2005) report, Joint Risk Assessment (JAR) report, publication at WHO websites, National Action Plan for Health Security (NAPHS), and East Africa Regional One Health Strategy [24-27]. Ministry of Health (MoH) granted access for the use of a line list for publication. We reviewed, cleaned, incorporated, and conducted a descriptive analysis of the Excel-based line list using IBM Statistical Package for Social Science Version 26.0 (IBM SPSS 26). For categorical variables like gender, patient outcome, symptoms, and risk factors (i.e. exposure to sick or dead animals, animal products, and proximity to places where abortions among domestic animals occurred). We obtained the mean, median, mode, and standard deviation for a continuous variable like age. We tabulated risk factors by case classification (i.e. negative and positive) in the bivariate analysis. Furthermore, we conducted Pearson´s chi-squared test using two-by-two table tests to determine the association between a risk factor and RVF case classification. The level of association was measured at a P<0.05 level of significance. For the qualitative review of the study, we used the van Wijnagaarden et al. strengths, weaknesses, opportunities, and threats analysis method [28]. We extrapolated, compiled, and reviewed information, which was related to outbreak response coordination, disease surveillance at human-animal ecosystem interface, integrated animal-human early warning, alert and response system (EWARS), national public health and veterinary laboratory diagnostic capacity. Besides, the review was made on the application of a multi-sectoral One Health approach from the available documents. We grouped the information into available systems and resources (strengths and weaknesses) and contextual factors, and stakeholder analysis (opportunities and threats).

**Ethical consideration:** administrative clearance for publication of this editorial was provided by the Ministry of Health of South Sudan and WHO (WHO e-Pub no: ePub-IP-00331583-EC) to publish the result. Confidentiality was ensured as the line list was anonymized, and data protection measures were employed to ensure the security of the data.

## Results

**Figure 1:**
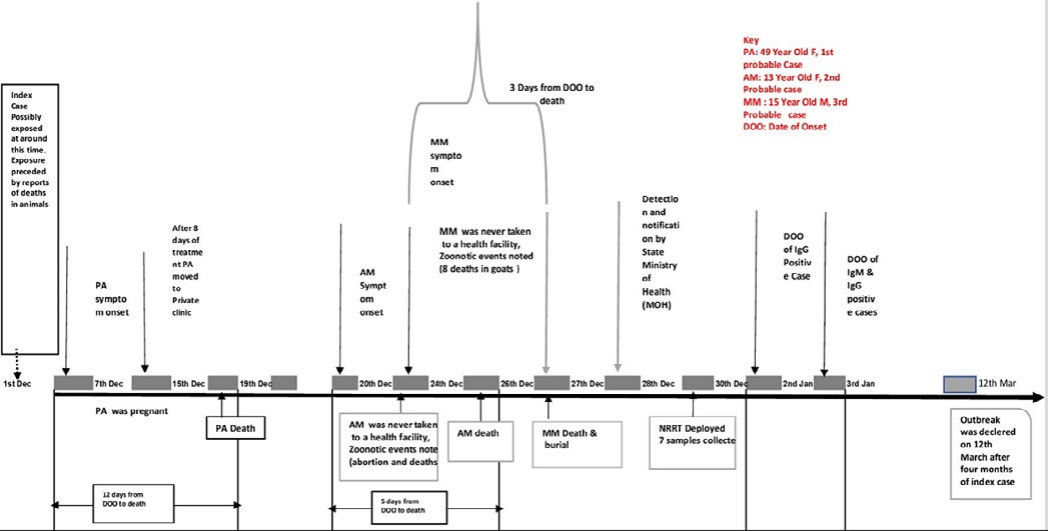
timeline of the Rift Valley Fever Outbreak, Yirol East county, South Sudan- December 2017 to April 2018

From December 2017 to December 2018, 58 suspected human RVF cases were reported and investigated by the rapid response teams (Figure 1). A total of four deaths were reported during the outbreak (case fatality rate (CFR) 40% (four deaths out of 10 cases (4/10)). The calculated attack rate was 55 cases per 100,000 population of Yirol East county from the population projection of Yirol East county was 102,158 in 2017. The males and females were equally affected (50%). The median age of the confirmed cases was 27 (Table 1). Four out of six (66.7%) confirmed cases had exposure to abortion or dead young domestic animal within the homestead (Table 1). During the field investigation of a total of three livestock were sick, of these two abortions were observed (one in goat, and one in sheep) and one bleeding in cattle. Two deaths were recorded in animals (one goat with tissue bleeding and one wide bird death)). A total of 28 domestic animal blood samples were tested for RVF antibody; of these 9 (32.1%) were tested positive. Out of 28 animal samples, nine were positive for RVFV antibody using ELISA (three were positive for IgM and six were positive for IgG). Of the total animal sample, 13 were negative for RVFV antibody (non-cases) and six were classified as a suspected case (the results were not known) (Table 2). The cases were reclassified based on laboratory and investigations results, such that as of 16th December 2018, there were a total of six (10.3%) laboratory-confirmed (one case was positive for both IgG + IgM, and five cases were positive for IgG). Three (5.2%) probable (samples were not collected), one (1.7%) suspected (result was not known), and 48 (82.8%) non-cases (negative for both IgM and IgG) were reported (Table 2).

**Table 1:** socio-demographic characteristics and epidemiological distribution of RVF in South Sudan-December 2017 to December 2018

Variable	Category	Confirmed cases (6) (%)	Probable cases (n=3) (%)	Suspected cases (n=16) (%)
Patient outcome	Alive	6(100%)	0(0.0%)	14(87.5%)
	Died	0(0.0%)	3(100.0%)	2(12.5%)
	Median	27	15	11
Gender	Female	3(50.0%)	2(66.7%)	38(50.7%)
	Male	3(50.0%)	1(33.3%)	37(49.3%)
Exposure to sick or dead animal	Yes	1(16.7%)	2(66.7%)	13(81.2%)
	No	5(83.3%)	1(33.3%)	3(18.7%)
Proximity to abortion or dead young domestic animals in homestead	Yes	4(66.7%)	1(33.3%)	13(81.2%)
	No	2(33.3%)	2(66.7%)	3(18.7%)

**Table 2:** summary of laboratory investigations, rift valley outbreak, South Sudan-December 2017-December 2018

Samples shipped to WHO/FAO collaborating laboratories	Number
Human	55
Livestock	28
**Total samples (both human and livestock)**	83
Human samples - PCR negative for Ebola, Marburg, CCHF, and RVF	48
Human samples - RVF serology - IgM and IgG positive (high titers)	1
Human samples - RVF serology - IgG positive (high titers)	5
Human sample-RVF serology-Not known	1
**Total human sample**	55
Livestock samples - RVF serology - IgM positive	3
Livestock samples - RVF serology - IgG positive	6
Livestock sample-RVF serology negative (both IgM and IgG)	19
**Total livestock sample**	28

The most frequent symptoms among the suspect, probable, and confirmed human cases were bleeding, fever, headache, joint pain, and muscle pain. Bleeding such as nasal bleeding, gum bleeding and petechial rash were reported in all suspected and probable cases. Three out of six (50%) confirmed cases had bleeding manifestation (Table 3). The main risk factors included in the line list were exposure to morbid or dead animals and proximity to animals that had an abortion or young domestic animals that died. Cases (confirmed, probable, and suspected) cases have 1.3 times has a higher risk of exposure than none cases. There was no significant difference in exposure to the sick or dead animal among RVF cases (confirmed, suspected, and probable) than non-RVF cases (P=0.68). The identified threats were lack of the availability of human and financial resources, challenging access to the communities, and political insecurity. During the RVF outbreak, the identified strengths included the existence of multi-sectorial outbreak coordination at the national level; availability of trained multi-disciplinary Rapid Response Teams (RRTs); the existence of national disease surveillance guidelines; availability of surveillance tools such as case definition; joint risk assessment and zoonotic disease prioritization; functional National Public Health Laboratory (NPHL) and Central Veterinary Diagnostic Laboratory (CVDL); and, the existence of partners institutes and organizations for multi-sectoral coordination. The weaknesses identified during the study were delayed case detection and notification, delayed outbreak announcement, lack of veterinary services, the weak linkage between animal and human disease surveillance systems, lack of clinical history taking and diagnostic capacity, shortage of laboratory reagents, and poor understanding and application of multi-sectoral one health coordination. The opportunities were the existence of a functional state ministry of health and state partners (non-Governmental and UN Organizations) for multi-sectorial coordination; the existence of the Early Warning, Alert and Response System (EWARS); the existence of the NPHL and CVDL molecular testing centre; and the existence of the Public Health Operation Center (PHEOC) for all public health hazard coordination (Table 4).

**Table 3:** list of symptoms among RFV cases, South Sudan-December 2017 to December 2018

Symptoms	Confirmed RVF (n=6)	Probable cases (n=3)
Bleeding	3(50.0%)	3(100.0%)
Breast pain	1(16.6%)	0(0.0%)
Chest pain	1(16.6%)	0(0%)
Convulsion	0(0.0%)	1(33.3%)
Cough	1(16.6%)	0(0.0%)
Dyspnoea	1(16.6%)	0(0%)
Fever	5(83.3%)	3(100.0%)
Headache	5(83.3%)	3(100.0%)
Joint pain	4(66.6%)	3(100.0%)
Muscle pain	2(33.3%)	3(100.0%)
Pain behind the eye	1(16.6%)	0(0.0%)
Sore throat	6(100%)	3(100.0%)
Vomiting	0(0.0%)	0(0.0%)

**Table 4:** (SWOT) analysis of Rift valley fever outbreak response, South Sudan-December 2017 and December to April 2018

Domain	Strengths	Weakness	Opportunities	Threats
Outbreak response coordination	1. Public health emergency operation center function to coordinate public health emergency response.	1. Weak public health coordination at the state level.	1. Availability of state ministry of health structures and leadership, and state health partners (non-governmental and UN Organizations).	1. The development of the national One Health approach strategy was not finalized, i.e. still at the draft stage.
	2. Trained multi-disciplinary rapid response team are available at the national state level	2. Take four months (December 2017 to March 2018) to declare an outbreak of RVF.	2. Availability of trained rapid response Team in 10 states of South Sudan.	2. Lack of multi-sectorial coordination at the state and county level.
				3. Multi-sectoral coordination at national level mostly attended by public health experts from human health,
				4. Bad roads network and insecurity that hinders deployment of outbreak investigation and response team.
Disease surveillance at animal-human ecosystem interface	1. The South Sudan national IDSR guideline listed RVF as one of reportable diseases.	1. Delayed detection, notification and confirmation of RVF cases (index case date of onset was 7^th^ December, notification 28^th^ December, confirmation on the 3^rd^ January 2018).	1. Availability of the IDSR guideline and surveillance tools.	1. Shortage of human and financial resources.
	2. Surveillance tools such as case definitions, and reporting formats were printed and distributed.	2. Failure to detect and notify livestock deaths prior to first human cases.	2. Existence of early warning, alert and response system (EWARS).	2. High turnover of trained health workers.
			3. Existence animal and human health implementing partners.	3. Weak collaboration among stakeholders at national and state level.
National diagnostic laboratory	1. There are functional national public health laboratory and central veterinary diagnostic laboratory.	2. Lack of reagents to confirm animal and human samples.	1. The National Public Health Laboratory has RT-PCR machine to confirm a number of viral hemorrhagic disease such as Ebola, Marburg, Yellow Fever, CCHF and RVF.	1. Lack of interest of donors and financial institute to support the laboratories.
		3. Lack of resources such as trained manpower, finance and supplies.	2. Existence of partners including the UN organizations.	2. Lack of motivation packages and salary to retain trained manpower at public health and veterinary laboratories.
				3. The laboratory results for the animal health took longer than expected
One Health approach	1. Incorporation of One Health approach into the South Sudan National Action Plan for Health Security (NAPHS) 2020-2024	3. Lack of clearly developed national One Health approach strategy.	5. Existence of partners at national and state level.	1. Both government and partners high staff turnover
	2. Joint risk assessment (JRA) was conducted for the prioritization of Zoonotic diseases	4. Irregular or no coordination among One Health approach implementing partners.		
			6. The IDSR guidelines prioritized a number of zoonotic disease for surveillance.	

## Discussion

The 2017 to 2018 outbreak in Yirol East County is the first major recorded RVF outbreak in South Sudan. From December 2017 and May 2018, South Sudan reported 25 human RVF cases. A total of five deaths were reported during the outbreak (case fatality rate (CFR) 20.0%). However, before the independence in 2011, South Sudan, as part of the White Nile Region of Sudan, experienced RVF outbreaks in 1973, 2007, and 2010. Therefore, there is a high possibility of cross-border circulation of the virus due to livestock movement, livestock trade, climate variability, and epizootic sweeping to South Sudan [13, 14]. Rift valley fever is mostly suspected when human cases appear which is not the ideal situation as outbreaks occur first in the animal population which should serve as an early warning for the human outbreak. In this outbreak, in the absence of veterinary services and lack of coordination between the veterinary and human public health sectors, the detection of human cases was delayed. The Yirol East County, South Sudan outbreak was detected in a dry season of 2017, 2018, yet RVF outbreaks largely follow flooding [29]. This is not the usual encounter, as it was noted by Hassan et al. (2011) [13]. Nevertheless, Payams of Yali and Pagarau in Yirol East county are surrounded by lakes and rivers which supply water for humans, livestock, and wildlife. This coupled with the wetland pools and stagnant water created by rainfall, established a favourable ground for mosquito breeding [30,31]. Besides, the area is typical of cattle keeping communities in South Sudan; where large swaths of designated cattle camps where young men tend to herd cattle moving around with the animals searching for pasture and drinking water. Hence, flooding in the geographic area with high-density wildlife and livestock created a favorable environment for RVFV transmission [32,33].

Rift valley fever outbreak is mostly suspected when human cases appear, which is not the ideal situation as outbreaks occur first in the animal population which should serve as an early warning for human outbreak [34]. In this outbreak, in the absence of veterinary services and weak coordination between the veterinary and human public health sectors, the detection of human cases was delayed by two months [22]. In the reported human cases, the epidemiological profile and clinical pattern of the cases were like what has been seen in other outbreaks [33,35]. The most common symptoms were fever and headache. As seen in the 2012 Mauritania RVF outbreak, the hemorrhagic manifestation was quite common (90%) in this outbreak, though most were epistaxis and bleeding of gums [36,38]. The case fatality ratio (CFR) of 20% is higher than the 1% CFR threshold, but similar findings of increased CFR were reported in Madagascar (3%) and Mauritania (50%) [37,38]. The high CFR is probably related to the underestimation of RVF cases due to the weak health system, weak community and health facility surveillance, lack of capacity to diagnose RVF, and poor health-seeking behavior. We saw an increased exposure to carcasses and raw milk consumption; 40.7% of cases reported exposure to the risk factors in our study. A study in South Africa revealed that 89% of confirmed RVF cases have contact with animal blood, tissues, or other body fluids [36]. Furthermore, the finding of our study is consistent with the findings in Tanzania’s and Kenya´s RVF outbreaks in 2007, whereby 40% of cases reported contact with sick animals and carcasses [3,9,10]. The finding entails focused risk assessment and risk communication interventions at the national and sub-national levels [25]. Rift Valley fever is one of the reportable zoonotic diseases; the Integrated Disease Surveillance and Response (IDSR) guidelines enable the national, state, and county health authorities to report immediately if RVF is suspected at the health facility and community level [39]. The guidelines incorporated tools, including RVF case definitions and reporting tools. A standard RVF case definition and other surveillance tools were used during the outbreak investigation [40]. However, surveillance systems and links at the animal-human ecosystem interface remained weak [22]. The situation was compounded by the weak health system, lack of human and financial resources, humanitarian situation, lack of coordination and leadership at various levels. Nevertheless, there are still capacities and success stories in South Sudan that will improve the RVF surveillance system; this includes s the decentralization of the IDSR/EWARS system and prioritization of zoonotic disease through joint risk assessment. Besides, there is an improved capacity at the NPHL to test for Ebola virus disease (EVD), RVF, Yellow Fever, Influenza virus, Marburg, and Coronavirus [41] using RT-PCR molecular diagnostic technique. The existence of partners such as FAO, Ministry of Livestock and Fishery and other partners will improve the multi-sectoral coordination of the One Health approach.

A total of 31 human blood sample and 28 animal blood sample was tested using ELISA and RT-PCR for RVF. There was a significant delay of confirmation of the outbreak, the reasons being delayed transportation of samples to reference laboratory and lack of laboratory capacity within the country. In 2019, South Sudan was able to test for RVF using RT-PCR as part of EVD preparedness but there is a strong need to increase the capacity of testing, especially the ELISA test to know the current and previous RVF infection [27]. Moreover, there is a need to establish a functional veterinary sector in the country for animal outbreak investigations and testing capacities. Furthermore, the deferential test for EVD, Marburg, CCHF, Yellow Fever yielded negative results, which is standard practice to exclude other differentials of disease presenting with a hemorrhagic clinical presentation [28]. A multi-sectoral task force established the outbreak response coordination. The task force was represented by the Ministry of Health, Ministry of Wildlife, Environment, Livestock and Fishery, and Defense. These were supported by partners including United Nations Children Fund (UNICEF), WHO, FAO, World Organization for Animal Health (OIE), Medicine Sans Frontier (MSF), European Union Humanitarian Aid, United States Agency for International Development (USAID), and health cluster partners. Though, the outbreak response plan was multi-sectoral certain aspects of the plan, such as the disease control at the animal-human interface, animal and entomological surveillance, was never funded and thus not implemented. Moreover, the community did not appreciate the lack of attention to animal health. During active case searches, the communities regularly asked why much attention was devoted to looking for sick persons, yet their domestic animals were dying every day, and nothing was being done for them. Often, the animal and the environmental component of one health are neglected or underfunded; hence, equitable inclusion of all component are very essential to fulfill the definitions of one health approach [42]. Furthermore, the absence of skilled veterinary personnel at the subnational level made it hard to constitute multidisciplinary teams to conduct active searches. To a community that highly values their cattle and other domestic animals, this was a “half measure response to their plight”. It has been demonstrated that the One Health approach can result in improved zoonotic disease surveillance at the animal-human-ecological interface; though, the implementation of the approach requires context analysis, political commitment, institutional and financial capacity [43].

## Conclusion

The outbreak imposed a challenge to both animal and public health in South Sudan. In addition to human losses, a significant loss of livestock was reported. From the study findings, it is evident that South Sudan lacks the basic capacity to respond to zoonotic disease outbreaks at the national and sub-national levels. There was delayed notification and investigation of the outbreak, delayed testing and result communication, and a lack of national capacity to confirm RVF. These were compounded by a lack of knowledge on the geographic distribution of RVF in the country. However, the outbreak was an important avenue to record challenges, gaps, and lessons to improve disease surveillance at the human-animal-environment interface, improve disease prevention and control, build testing capacity, and improve collaboration and partnership. We have drawn practical recommendations critical to improving RVF prevention and control in South Sudan. The recommendations include: improve zoonotic disease detection and reporting through enhanced disease surveillance and laboratory capacity; draw lessons from other countries´ experiences to establish efficient RVF control and prevention strategy; establish multi-sectoral One Health platform; and undertake a seroprevalence study in human and animal covering the wide geographic area.

### What is known about this topic

- Rift Valley fever (RVF) is one such zoonotic disease that is endemic in parts of Africa and is known to infect a diverse group of animals, including cattle, goats, sheep, buffalos, camels, and others most human infections result from direct or indirect contact with the organs or blood of infected animals. The virus can be transmitted to humans by handling animal tissue during slaughtering or butchering, assisting with animal births, conducting veterinary procedures, or the disposal of carcasses or fetuses;
- Certain occupational groups such as herders, farmers, slaughterhouse workers, and veterinarians are at higher risk of infection;
- One Health approach (multi-sectoral) is required to contain RVF outbreaks and mitigate their impacts.

### What this study adds

- In the absence of strong surveillance systems in the animal health sector, most zoonoses are only detected and confirmed when they cross to humans. However, for cattle keeping communities like in South Sudan, a response that is entirely focused on human health does not meet the needs of the community that continue to suffer economic losses;
- In malaria-endemic countries; syndromic malaria surveillance and symptomatic treatment of malaria cases can mask an RVF outbreak, especially during the malaria peak seasons (rainy season in South Sudan). Laboratory confirmation of malaria cases is critical, and multiple cases of fever with negative malaria tests can allow testing for other causes of fever such as RVF and Dengue;
- South Sudan lacks the basic capacity to investigate and respond to Rift Valley fever outbreaks. There is no one health policy, strategy or mechanism established to handle health events at the human-animal-environmental interface.
